# Observing a trained demonstrator influences associative appetitive learning in rats

**DOI:** 10.1098/rsos.221224

**Published:** 2023-04-12

**Authors:** Laura A. Agee, Miriam E. Ortega, Hongjoo J. Lee, Marie-H. Monfils

**Affiliations:** Department of Psychology, The University of Texas at Austin, 108 E. Dean Keeton Stop A8000, Austin, TX 78712-1043, USA

**Keywords:** social learning, appetitive social learning, associative learning, observational learning

## Abstract

The ability to acquire information about the environment through social observation or instruction is an essential form of learning in humans and other animals. Here, we assessed the ability of rats to acquire an association between a light stimulus and the presentation of a reward that is either hidden (sucrose solution) or visible (food pellet) via observation of a trained demonstrator. Subsequent training of observers on the light-reward association indicated that while observation alone was not sufficient for observers to acquire the association, contact with the reward location was higher in observers that were paired with a demonstrator. However, this was only true when the light cue predicted a sucrose reward. Additionally, we found that in the visible reward condition, levels of demonstrator orienting and food cup contact during the observation period tended to be positively correlated with the corresponding behaviour of their observer. This relationship was only seen during later sessions of observer training. Together, these results suggest that while our models were not sufficient to induce associative learning through observation alone, demonstrator behaviour during observation did influence how their paired observer's behavioural response to the cue evolved over the course of direct individual training.

## 1. Introduction

The ability to acquire information through observation of, or instruction by, a conspecific, i.e. social learning, is a trait that has been observed in a variety of species both social [1–4] and traditionally solitary [5,6]. Social learning is a particularly advantageous way of acquiring information about the environment in that it allows individuals to learn about potential new resources or threats without having to undergo the potentially dangerous (or deadly) process of examining unknown stimuli themselves. In humans, social learning is taken to the extreme, with an individual's ability to function and survive in modern society being largely contingent on their ability to communicate with and learn from the others around them. As such, dysfunction in the social learning process or the cognitive abilities underlying these processes (e.g. imitation, social attention or implicit social reward) can be harmful to individual function and may be relevant to disorders with a social component such as autism spectrum disorders [7–11].

Understanding the neurobiological processes that support social learning is an important step in dealing with dysfunction in that system. While studies using human models of social learning have been hugely informative, the development of non-human animal models of social learning remains necessary for detailed study of the neural circuitry and neurochemical processes underlying the social acquisition of information. The development of rodent models of fear-motivated observational social learning of both operant behaviours [12–14] and associative relationships [15–18] has been largely successful. By contrast, while the development of rodent models of observational forms of reward-motivated social learning has seen some success in social learning models of operant foraging behaviour [19–21], the social transmission of food preference (STFP) paradigm [1,22] remains the best analogue of a reward-based associative model of social learning.

In the STFP paradigm, an ‘observer' animal interacts with a ‘demonstrator', a conspecific that has recently eaten a novel food, and subsequently acquires a preference for consuming said food. This model hinges on the innate tendency for rats [1], mice [2] and other rodents [23,24] to form a preference to a diet that has had its scent paired with carbon disulfide (CS_2_), a chemical present in a nasal cavity of rodents [25,26]. While STFP is a fascinating form of social learning in its own right, a number of characteristics suggest that it may not be the best model of social observational associative learning. Specifically, observers in the STFP paradigm are insensitive to virtually all characteristics of their demonstrators, including demonstrator age [27], reliability [29], relevance [30], familiarity or kinship [31,32], and even whether the demonstrator was experiencing acute gastrointestinal distress during the interaction ([33,34]; though see also [35]) (for review, see [36]). Even more strikingly, rodents are capable of acquiring food preferences both while anesthetized [37] and without a conspecific present, as long as a scent is paired with CS_2_ [25,26], an effect that is unimpaired by past pairing of CS_2_ with aversive stimuli [38]. Furthermore, demonstrator characteristics such as familiarity/kinship [14,16,17,32], species strain [39] and dominance [18] all affect other forms of observational learning. Taken all together, this would suggest fundamental differences between STFP and observational forms of social learning. Moreover, these results suggest that limited or no conscious processing of exterior stimuli is involved in the STFP learning process (see [28] for review).

In the experiments described here, we aimed to develop a workable model of explicitly observational associative appetitive learning. We tested the effect of pre-conditioning observation of a trained demonstrator on rats' ability to acquire an association between a neutral light cue (the conditioned stimulus, CS) and the presentation of a rewarding unconditioned stimuli (US) in the form of either a hidden reward (a bottle of sucrose solution) or a visible reward (plain snack pellets delivered to a clear cup). In order to rule out enhanced acquisition of the association by socially induced stimulus enhancement of the CS, we also scored the amount of time observers spent attending to the cue during their training. We hypothesized that if observational learning were occurring, observers with trained demonstrators would display above-baseline levels of approach to the reward site after CS presentation but before US availability during individual acquisition immediately after observation (i.e. at session 1 of individual learning).

## 2. Methods

All experiments were conducted in compliance with the National Institutes of Health Guide for the Care and Use of Experimental Animals and were approved for use by The University of Texas at Austin Animal Care and Use Committee.

### 2.1. Experimental overview

#### 2.1.1. Experiment 1: observational associative learning with a hidden reward

**Figure 1.**
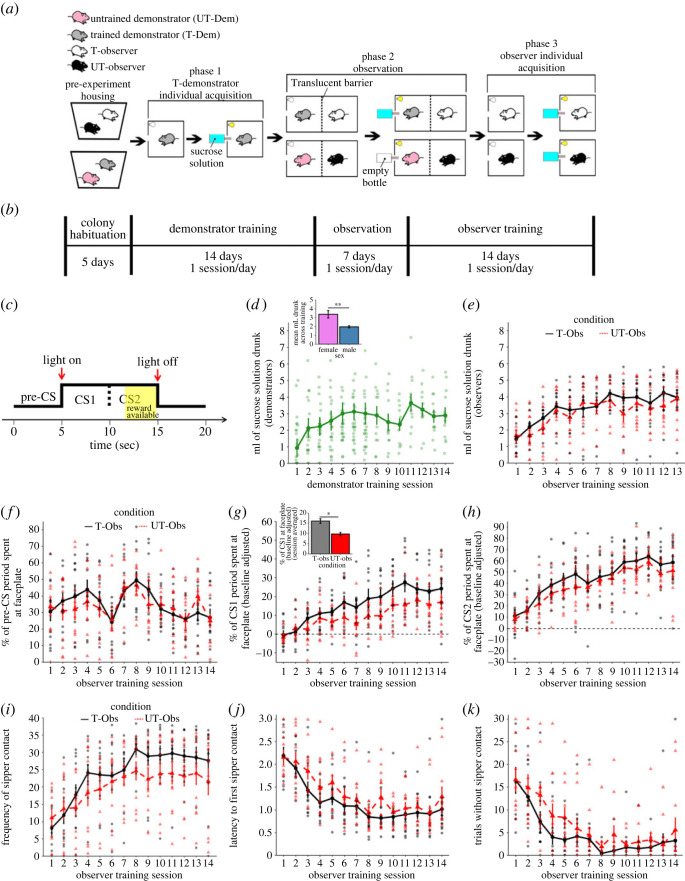
Experiment 1 behavioural design and results. A graphical depiction of (*a*) the treatment of the rats in the various conditions, (*b*) the timeframe of each experimental phase, and (*c*) how the stimulus presentation was divided up for scoring. Mean (+/− s.e.m.) consumption of the sucrose solution over the course of (*d*) demonstrator and (*e*) (line graph) observer training suggested successful acquisition of the CS–US association. Additionally, (*d*) (bar graph) a comparison of drinking averaged across all sessions by sex shows significantly higher sucrose consumption in female demonstrators as compared with male demonstrators. (*f*–*h*) Contact with the faceplate through which the sucrose solution was made available was examined between T-Obs rats and UT-Obs rats across sessions during the (*f*) pre-CS, (*g*) (line graph) baseline (i.e. pre-CS) adjusted CS1, and (*h*) baseline adjusted CS2 timepoints. (*g*) (bar graph) An overall effect of experimental condition was detected on faceplate contact during the CS1 period, with T-Obs rats spending a significantly higher amount of time in contact with the faceplate than UT-Obs rats across sessions. No overall effect of condition was detected on (*i*) the average frequency of sipper contact per trial, (*j*) the average latency to first sipper contact per trial, or (*k*) the number of trials in which no sipper contact was made in a session. * p < 0.05, ** p < 0.01*.*

(See figure 1*a,c* for a graphical overview of experiment 1.)

In experiment 1, the appetitive conditioning paradigm was composed of the presentation of a light CS followed by the presentation of the spout to a bottle containing sucrose solution. Rats were only able to access the bottle by sticking their snout through a small hole in the conditioning chamber. The side of the chamber through which the bottle was inserted was opaque, making it impossible to see what was being presented. Rats were assigned to one of four conditions:

1) Trained demonstrator (T-Dem): rats assigned to the T-Dem condition went through standard associative learning procedures (14 sessions with 30 trial presentations of the CS–US pairing, see Apparatus and stimuli section for additional details) prior to serving as a demonstrator in the experiment (Phase 1, demonstrator training phase). A day prior to their associative learning training, T-Dem rats were allowed an hour to habituate to the conditioning chamber with free access to the sucrose solution to help them overcome any neophobia.
2) Untrained demonstrator (UT-Dem): rats assigned to the UT-Dem condition did not receive associative conditioning prior to serving as a demonstrator, although they were allowed the same chamber habituation as T-Dem rats. Additionally, UT-Dem rats were put through a single session of the standard training protocol with the exception that no bottle of sucrose solution was provided. This was to habituate them to the CS and the sound of the bottle assembly inserting the spout of the bottle. Other than these 2 days of habituation, UT-Dem rats were left undisturbed throughout Phase 1 of the experiment. UT-Dem rats were not provided with unpaired CS–US presentations to avoid the development of disruptive behaviours.
3) Observer with trained demonstrator (T-Obs): T-Obs rats were paired with a same-sex rat assigned to the T-Dem condition. T-Obs rats were placed in the conditioning chamber with a translucent barrier separating them from the T-Dem rat and were allowed to observe the T-Dem rat undergo seven sessions of the standard sucrose appetitive conditioning protocol (Phase 2, observation phase). Following Phase 2, T-Obs rats underwent the same training procedure as T-Dem rats (Phase 3, observer individual acquisition). Twenty-four hours separated each phase of the experiment. Past research which successfully produced social learning would suggest that this 24 h period separating the end of observation and the beginning of individual learning is sufficient for consolidation to occur and for socially learned behaviours to manifest [12,15].
4) Observer with untrained demonstrator (UT-Obs): UT-Obs rats were treated the same as T-Obs rats with the exception that during Phase 2 they were (i) paired with a rat assigned to the UT-Dem condition, and (ii) their paired UT-Dem rat was run through the standard conditioning procedure with an empty bottle rather than a bottle filled with sucrose solution.

#### 2.1.2. Experiment 2: observational associative learning with a visible reward

**Figure 2.**
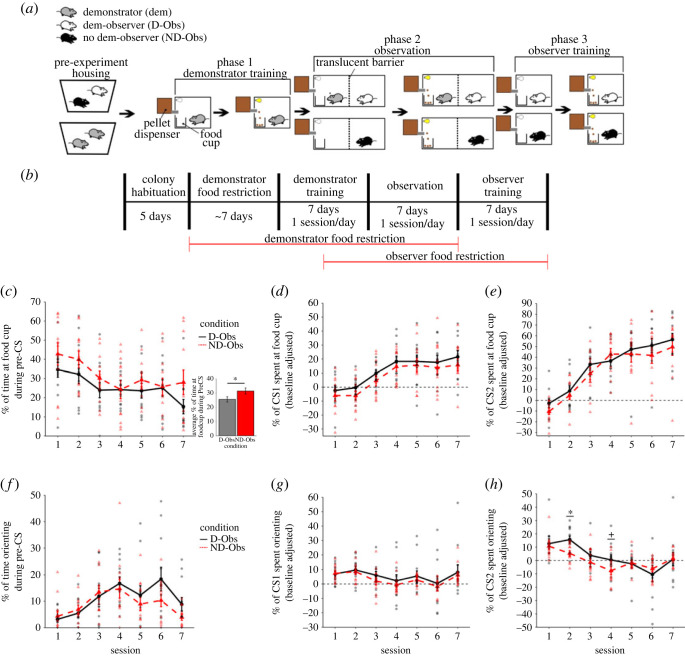
Experiment 2 behavioural design and results. (*a*) A graphical depiction of the treatment of the rats in each in the conditions in experiment 2 and (*b*) the timeframe of each experimental phase and food restriction. An examination of food cup contact found that (*c*) during the pre-CS period, an overall reduced time of food cup contact was observed in D-Obs rats (bar graph) which did not differ across sessions (line graph). By contrast, no differences in food cup contact were observed between D-Obs and ND-Obs rats during the (*d*) CS1 and (*e*) CS2 period. Similarly, no significant difference between the experimental conditions was seen in orienting to the cue during the (*f*) pre-CS and (*g*) CS1 period. However, during the (*h*) CS2 period, D-Obs rats displayed slightly higher orienting at sessions 2 and 4, with the latter session not quite reaching significance. *p < 0.05*, +p <* 0.1*.*

(See figure 2*a*,*b* for a graphical overview of experiment 2.)

In experiment 2, the appetitive conditioning paradigm used standard snack pellets deposited into a clear glass jar as the US. As before, a light cue was used at the CS, the only difference being that it was located on top of the conditioning chamber. Details of the conditioning and observation sessions can be found in the Apparatus and stimuli section below. Rats in this experiment were assigned to one of only three conditions:

1) Demonstrator (Dem): rats assigned to the Dem condition were given a single session of habituation to the chamber, food cup and food pellets before undergoing seven sessions of conditioning during Phase 1 (demonstrator training phase) of this experiment.
2) Observer with demonstrator (D-Obs): rats assigned to the D-Obs condition were paired with a same-sex rat assigned to the Dem condition. During Phase 2 (observation phase) of the experiment, they were separated from their demonstrator by a translucent barrier and allowed to observe the behaviour of their Dem rat over the course of seven sessions of standard appetitive conditioning training. In Phase 3 (observer training phase) observation period, D-Obs rats were put through the same seven-session conditioning procedure that Dem rats had undergone. As in experiment 1, 24 h separated each phase of the experiment.
3) Observer with no demonstrator (ND-Obs): ND-Obs rats were treated identically to D-Obs rats with the exception that during Phase 2 of the experiment, no demonstrator was placed in the cage with them (the translucent barrier separating them from the food cup and light remained). Phase 2 observation sessions otherwise went on as described. As such, while ND-Obs rats did see food pellets deposited into the jar, there was no demonstrator present to consume them or react to the CS.

Notably, neither ND-Obs or D-Obs rats were given pre-observation access to the reward pellets. As such, it is possible that they did not recognize them as palatable.

### 2.2. Subjects

#### 2.2.1. Experiment 1: observational associative learning with a hidden reward

Subjects were 48 Long-Evans rats, 24 females and 24 males, obtained from Harlan (Houston, TX, USA) at roughly nine weeks of age (*n* = 12/condition with 6/sex in each condition), with T- and UT-Obs rats arriving about three weeks after T-Dem and UT-Dem rats. All rats were housed in same-sex dyads at arrival with T-Obs assigned rats always housed with a UT-Obs assigned rats and T-Dem assigned rats always housed with a UT-Dem assigned rat. This separation of observers from their demonstrators was to avoid forming a socially transmitted food preference for the reward prior to individual learning. All rats were allowed *ad libitum* food and water access throughout the experiment. All subjects were kept on a 12 h light cycle (8.00 to 20.00 lights off) with all experimental procedures taking placing during their dark cycle.

#### 2.2.2. Experiment 2: observational associative learning with a visible reward

Observers from experiment 1 were re-used as demonstrators in experiment 2 (*n* = 12 demonstrators with 6/sex). Experiment 2 observers were male (*n* = 12, 6/observer condition) and female (*n* = 12, 6/observer condition) Long-Evans rats obtained from Harlan at nine weeks of age. Rats were kept on the same light cycle and in the same colony as rats from experiment 1. Prior to training, rats were food restricted to 90% of their free feeding weight and maintained at that weight throughout the experiment. During food restriction, all rats were weighed daily. Demonstrator food restriction began 7 days prior to their first training session, while observer food restriction began 3 days prior to their first observation session.

### 2.3. Apparatus and stimuli

#### 2.3.1. Experiment 1: observational associative learning with a hidden reward

All phases of experiment 1 took place in a Med Associates Inc. (Fairfax, VT, USA) conditioning chamber (30.5 × 24.1 × 29.2 cm, L×W×H) with a steel grid floor housed inside a sound-attenuating isolation cubicle. Chambers were affixed with cameras (KT&C USA, Fairfield, NJ) filming the chamber from above as well as an exhaust fan. Each chamber was Med-PC controlled and equipped with a retractable bottle assembly, a white house light, and a second light used as the CS. When inserted, bottle spouts were reachable through a small hole in the wall on the side of the chamber approximately 8.5 cm above the floor. The light CS was located at the other end of the same wall at roughly the same elevation. For sucrose conditioning, bottles (50 ml Falcon tube sealed with a sipper plug) were filled with 30 ml of a 0.5 M sucrose/water solution, the concentration which has previously been found to be most favoured by rats [40]. A small plate was placed under every bottle to catch any drip. The volume from each bottle and any liquid on the drip plate was measured at the end of each session. During the observation phase of the experiment, barriers constructed from flexible, clear 40-gauge vinyl sheets with aluminium sheet metal as siding were secured at the centre of the conditioning chamber and kept in place using Velcro strips. The barriers kept observers and demonstrators from interacting physically in any way, while allowing the observer to maintain visual access to their demonstrator.

A single sucrose appetitive conditioning procedure was roughly an hour long and involved 30 CS–US pairings (30 trials), with a variable inter-trial interval averaging 90 s (range 60–120 s), and with the first presentation occurring 40 s after the start of the protocol. For each trial, the light CS was turned on for 10 s. Seven seconds into the CS presentation, the bottle was inserted into the sipper hole and made available for 3 s. Bottle availability and CS presentation co-terminated at the end of the trial. This appetitive conditioning procedure was the same at all phases of the experiment, with the exception that during the UT-Obs observation phase the inserted bottle was left empty to prevent UT-Dem rats from acquiring the CS–US association. Training sessions occurred once per day, with the demonstrator training phase lasting 14 sessions, the observation phase lasting seven sessions, and the observer training phase lasting 14 sessions.

#### 2.3.2. Experiment 2: observational associative learning with a visible reward

All phases of experiment 2 took place in standard Habitest conditioning chambers from Coulbourn Instruments (Whitehall, PA, USA) (25.5 × 30.5 × 30.5 cm, L × W × H). Floors were composed of stainless-steel rods (0.5 cm in diameter, spaced 1.0 cm apart), and each chamber was enclosed in a sound-attenuating isolation box (Coulbourn Instruments; 61 × 58.4 × 46 cm, L × W × H). Video cameras were mounted at the back of each box and angled such that a full view of the box was available. The original pellet receptacle was a metal bin positioned on the right-hand wall of the chamber, directly across from the camera. Its entrance was approximately 2.5 cm above the floor and flush with the wall. In order to make sure that the grain pellets (45 mg, TestDiet, Richmond, IN, USA) that were serving as the food reward (US) were visible to the observer, the original food receptable was modified with a metal flap covering a section of narrow PVC piping attached to the food dispenser with a section of flexible soft-plastic piping. A 4 oz hexagonal clear glass jar was secured to the chamber floor with velcro and positioned to receive the pellets ejected from the PVC pipe. Unlike in the sucrose experiment, the house light was used as the CS and was otherwise left off for the duration of the experiment. Enough light did filter in from outside the isolation chamber to allow the observer to see their demonstrator and the food cup even in the absence of the CS. The CS was a bright, white light that was positioned at the centre-top of the same wall as the original pellet receptacle and illuminated the entire chamber when on. During observation sessions, D- and ND-Obs rats were separated from the side of the chamber containing the food cup and CS (and the demonstrator, in the case of the D-Obs rats) using a clear barrier constructed from the same components and secured in the same way as described above for experiment 1.

Each training session consisted of 15 CS–US pairings (trials). The house light was illuminated for a period of 10 s, 7 s into which three food pellets (1 pellet/second) were ejected into the glass jar. Trials were separated by a variable inter-trial interval (ITI) averaging roughly 120 s, with 60 s, 120 s and 180 s being the three candidate ITI lengths. ITI lengths were sampled with replacement, so total session length varied from approximately 30 to 40 min in duration. Demonstrator training, observation and observer training phases all consisted of seven sessions of training, with one session of training occurring per day. Prior to demonstrator training, Dem rats were habituated to the chamber, the food pellets and the food cup during a 30 min long habituation session in which they were placed in the chamber and a single pellet was delivered to the food cup every 2 min.

### 2.4. Behavioural scoring

#### 2.4.1. Sucrose conditioning scoring

Observer training sessions scores were taken from the video recordings of each session by an observer blind to the experimental condition of each rat. Due to video loss, observation sessions and demonstrator training sessions were not able to be scored. End-session sucrose solution volumes confirmed that demonstrators had acquired the CS–US association. For observer individual training sessions, faceplate contact was scored for any time that a rat made direct contact with the faceplate, which was a small square of metal surrounding the hole in which the sucrose bottle was accessible during training. Behavioural scoring for each trial was broken into three phases: (i) pre-CS, the 5 s before the light CS turned on, (ii) CS1, the first 5 s that the light CS was on, and (iii) CS2, the second 5 s that the light CS was on, including the final 3 s in which the sucrose sipper was available (figure 1*c*). Scores for each session were the average per cent of time engaging in a given behaviour across trials during each phase of scoring. In order to control for baseline differences in the amount of time spent examining the faceplate or CS, final scores for CS1 and CS2 were determined by subtracting out the average per cent of the pre-CS period spent engaging in a behaviour across the session. Rate of learning for each session was estimated by taking the difference between the response for a given session from the response at the previous session. Rate of learning was calculated separately for each timepoint. In addition to the manually scored behaviour, the frequency of sipper contact, number of trials without sipper contact, and the latency to first sipper contact were all scored automatically by the Med-PC system. For all automatic measures, there was data loss for *n* = 4 subjects at session 1.

#### 2.4.2. Food cup conditioning and observation scoring

All observer training sessions and all observation sessions for D-Obs rats were scored for experiment 2. As demonstrator's behaviour during training was not comparable to the observer's behaviour due to the demonstrator's previous conditioning experience, behaviour from the demonstrator training sessions was not scored. The demonstrator's behaviour was only scored for during the observation phase. Videos were scored by an observer blind to the condition of each subject in the observer training session and blind to subject identities in the scoring of observation sessions. The two behaviours scored for were: food cup contact and orienting. Food cup contact was scored for whenever a rat was making direct contact with the glass jar, food pellets were retrieved with their forepaws, face or mouth or when they had their head inside the jar. Orienting was scored for whenever a rat was rearing (standing on hind legs with front paws fully off the ground and not on the food cup) and facing in the direction of the light cue. The orienting score was determined by the duration of rearing, not the number of rears. Behavioural scoring for each trial was separated into a pre-CS, CS1 and CS2 period as described for sucrose conditioning. Final session scores were expressed as the per cent of each scoring period that a rat spent engaged in a given behaviour. Pre-CS averages for each behaviour were again subtracted out from the averages of CS1 and CS2 to control for potential baseline differences in the amount of time spent orienting to the cue or examining the food cup. Rate of learning was calculated for in the same way as was described for sucrose conditioning.

## 3. Results

All data analysis was completed in the R coding software with the Rstudio integrated development environment. Unless otherwise mentioned, analyses of differences in observer performance in both experiments and for all dependent measures were completed using a three-way mixed ANOVA with experimental condition and sex as between-subject factors and experimental session as the within-subject factor. Demonstrator acquisition and performance data were assessed using a two-way mixed ANOVA with sex as the between-subjects variable and session as the within-subjects variable. In the case that assumptions of sphericity were violated, the Huynh–Feldt epsilon was applied to the degrees of freedom as a correction. A *p* < 0.1 was set as the cut-off for *post hoc* testing and *p* < 0.05 was set as the cut-off for a result to be considered of general significance. *Post hoc* testing was completed by *t*-test pairwise comparisons of estimated marginal means and the Tukey method of adjustment for family-wise error rates where necessary. If the subset data did not meet *t*-test assumptions, a Mann–Whitney *U* test was run as an alternative. Effects sizes are reported as Cohen's d for parametric pairwise comparisons and *r* for non-parametric pairwise comparisons.

### 3.1. Experiment 1: observational associative learning with a hidden reward

#### 3.1.1. Sucrose drinking results

A mixed ANOVA with ml drunk over the course of demonstrator training as the dependent variable, session as the within-subjects variable and sex as the between-subjects variable confirmed that sucrose drinking increased across sessions (*F*_13,130_ = 2.416, *p* = 0.006) while also detecting a significant effect of sex (*F*_1,10_ = 10.352, *p* = 0.009, d = 1.86)—with females generally drinking more than males—but no interaction between the two factors (*F*_13,130_ = 1.186, *p* = 0.296) (figure 1*d*). To test whether male and female demonstrators were equally effective and to confirm that demonstrator performance was similar across observation sessions, a mixed ANOVA with ml drunk as the dependent variable, session as the within subject variable, and sex as the between subject variable was run. No effect of sex (*F*_1,10_ = 1.196, *p* = 0.192) or session (*F*_6,60_ = 0.687, *p* = 0.66) and no interaction between the two (*F*_6,60_ = 0.5, *p* = 0.806) was detected. Notably, however, demonstrator drinking did decrease substantially during the observation sessions (mean = 1.91 ml drunk, s.d. = 0.83) as compared with the final session of training (mean = 2.9 ml drunk, s.d. = 1.59), probably due to the observer serving as a distraction to the demonstrator. In the observer data, sucrose volume data were lost for a number of rats at session 2 (*n* = 8). Lost values were estimated for these rats using the average of the volume drunk in their first and third trials. Session 14 was not included in the analysis due to more complete data loss. Notably, residuals were slightly left skewed, resulting in a violation of normality assumptions that could not be corrected by data transform. As we were unable to identify any reliable non-parametric alternatives, we will proceed with the analysis, but results should be taken with some caution. Our ANOVA found a significant overall effect of session (*F*_12,240_ = 15.44, *p* < 0.0001), confirming increased sucrose consumption with task acquisition (figure 1*e*). No effect of sex (*F*_1,20_ = 0.468, *p* = 0.51) or condition (*F*_1,20_ = 0.661, *p* = 0.426), and no interactions between sex and condition (*F*_1,20_ = 0.05, *p* = 0.82), sex and session (*F*_12,240_ = 0.75, *p* = 0.686), or condition and session (*F*_12,240_ = 0.969, *p* = 0.475), were detected. A marginal three-way interaction between all variables were suggested by our results (*F*_12,240_ = 1.66, *p* = 0.085), but *post hoc* testing found no significant differences at any timepoint (all *p* > 0.1).

#### 3.1.2. Faceplate contact results

Our ANOVA of pre-CS faceplate contact found a significant effect of session (*F*_13,260_ = 6.658, *p* < 0.0001) and a significant three-way interaction effect between sex, condition and session (*F*_13,260_ = 2.672, *p* = 0.015), though this interaction did not survive *post hoc* testing (all *p* > 0.1) (figure 1*f*; electronic supplementary material, figure S1*a*–*c*). We found no effect of sex (*F*_1,20_ = 1.535, *p* = 0.23) or condition (*F*_1,20_ = 0.074, *p* = 0.789) and no interactions between sex and condition (*F*_1,20_ = 0.073, *p* = 0.79), sex and session (*F*_13,260_ = 0.538, *p* = 0.794), or condition and session (*F*_13,260_ = 1.056, *p* = 0.394) for the pre-CS period. For faceplate contact during the CS1 period, we found a significant effect of session (*F*_13,260_ = 17.372, *p* < 0.0001) and condition (*F*_1,20_ = 5.535, *p* = 0.029), but only a marginally significant effect of sex (*F*_1,20_ = 3.324, *p* = 0.083). No interactions between sex and condition (*F*_1,20_ = 1.803, *p* = 0.194), sex and session (*F*_13,260_ = 1.425, *p* = 0.194), session and condition (*F*_13,260_ = 1.154, *p* = 0.331), and no three-way interaction (*F*_13,260_ = 0.836, *p* = 0.567) were detected (figure 1*g*; electronic supplementary material, figure S1*d–f*). Lastly, our ANOVA of faceplate contact at CS2 found a significant effect of session as expected (*F*_13,260_ = 30.974, *p* < 0.0001), though, notably, our CS2 residuals did violate normality assumptions due to a slight left skew. No significant overall effects of condition (*F*_1,20_ = 1.389, *p* = 0.252) or sex (*F*_1,20_ = 1.46, *p* = 0.24), and no interactions between condition and sex (*F*_1,20_ = 0.549, *p* = 0.468), sex and session (*F*_13,260_ = 1.61, *p* = 0.122), condition and session (*F*_13,260_ = 0.301, *p* = 0.968), or condition, sex and session (*F*_13,260_ = 0.979, *p* = 0.456) were detected (figure 1*h*; electronic supplementary material, figure S1*g*–*i*).

#### 3.1.3. Sipper contact results

Our analysis of average latency to sipper contact per trial found only a significant effect of session (*F*_13,208_ = 29.46, *p* < 0.0001). No effects of condition (*F*_1,16_ = 2.42, *p* = 0.14) or sex (*F*_1,16_ = 2.82, *p* = 0.13) on latency were detected. Additionally, no condition by sex *F*_1,16_ = 0.54, *p* = 0.47), sex by session (*F*_13,208_ = 1.21, *p* = 0.27), condition by session (*F*_13,208_ = 0.845, *p* = 0.61), or three-way interactions (*F*_13,208_ = 1.54, *p* = 0.11) were detected (figure 2*i*; electronic supplementary material, figure S2*a*–*c*). Our results examining the average frequency of sipper contact per trial again found an effect of session (*F*_13,208_ = 20.11, *p* < 0.0001) and also detected an interaction between condition and session (*F*_13,208_ = 2.14, *p* = 0.03) as well as a marginal three-way interaction (*F*_13,208_ = 1.85, *p* = 0.065). Neither interaction survived *post hoc* testing (all *p* > 0.05). No overall effect of condition (*F*_1,16_ = 1.21, *p* = 0.29) or sex (*F*_1,16_ = 0.84, *p* = 0.37) were detected, and no interactions between sex and condition (*F*_1,16_ = 1.24, *p* = 0.25) or sex and session (*F*_13,208_ = 1.24, *p* = 0.25) were found (figure 2*j*; electronic supplementary material, figure S2*d*–*f*). Finally, our analysis of the total number of trials in which no contact was made with the sipper (omissions) found a significant effect of session (*F*_13,208_ = 31.36, *p* < 0.0001) and a significant three-way interaction (*F*_13,208_ = 2.82, *p* = 0.001). *Post hoc* testing found significant differences between trained and untrained females at sessions 3 (*Z* = 2, *p* = 0.04, *r* = 0.58), 5 (*Z* = 2.12, *p* = 0.034, *r* = 0.6) and 8 (*Z* = 2.2, *p* = 0.028, *r* = 0.636). Trained and untrained males were only significantly different at session 9 (Z = 2.09, *p* = 0.04, *r* = 0.6). No overall effect of condition (*F*_1,16_ = 2.17, *p* = 0.16) or sex (*F*_1,16_ = 1.92, *p* = 0.19), and no interaction between sex and condition (*F*_1,16_ = 0.06, *p* = 0.81), sex and session (*F*_13,208_ = 1.45, *p* = 0.14), or condition and session (*F*_13,208_ = 0.93, *p* = 0.52) (figure 2*k*; electronic supplementary material, figure S2*g*–*i*) were found.

### 3.2. Experiment 2: observational associative learning with a visible reward

#### 3.2.1. Food cup contact results

Our ANOVA of pre-CS food cup contact found a significant effect of session (*F*_6,120_ = 4.4, *p* = 0.0005) and a marginally significant overall effect of condition (*F*_1,20_ = 4.34, *p* = 0.05) that *post hoc* testing confirmed (*Z* = 2.021, *p* = 0.043), with ND-Obs rats tending to interact more with the food cup during the pre-CS period than D-Obs rats. No significant effect of sex (*F*_1,20_ = 1.915, *p* = 0.182) was detected, and no interactions between sex and condition (*F*_1,20_ = 0.277, *p* = 0.604), sex and session (*F*_6,120_ = 0.53, *p* = 0.78), condition and session (*F*_6,120_ = 0.53, *p* = 0.78), or condition, sex and session (*F*_6,120_ = 0.92, *p* = 0.48) were found (figure 2*c*; electronic supplementary material, figure S3*a*–*c*). Our ANOVA analysing food cup contact during the CS1 period found a significant effect of session (*F*_6,120_ = 18.62, *p* < 0.0001), but no effect of sex (*F*_1,20_ = 0.27, *p* = 0.61) or condition (*F*_1,20_ = 1.33, *p* = 0.26). Additionally, no sex and condition (*F*_1,20_ = 0.07, *p* = 0.8), sex and session (*F*_6,120_ = 0.89, *p* = 0.5), condition and session (*F*_6,120_ = 0.07, *p* = 1), or condition, sex and session (*F*_6,120_ = 0.57, *p* = 0.75) interactions were detected (figure 2*d*; electronic supplementary material, figure S3*d*–*f*). Finally, our ANOVA examining food cup contact during the CS2 period found an overall effect of session (*F*_6,120_ = 49.2, *p* < 0.0001), but no effect of sex (*F*_1,20_ = 1.47, *p* = 0.24) or condition (*F*_1,20_ = 0.73, *p* = 0.4) and no interactions between sex and condition (*F*_1,20_ = 0.09, *p* = 0.77), sex and session (*F*_6,120_ = 0.82, *p* = 0.55), condition and session (*F*_6,120_ = 0.69, *p* = 0.66), or between all three variables (*F*_6,120_ = 0.71, *p* = 0.64) (figure 2*e*; electronic supplementary material, figure S3*g*–*i*). Similarly, while our ANOVAs examining rate of change in food cup responding found significant effects of session at the CS1 (*F*_5,100_ = 2.66, *p* = 0.035) and CS2 (*F*_5,100_ = 3.96, *p* = 0.003) timepoints, no other effects or interactions were detected (all *p* > 0.1).

#### 3.2.2. Orienting results

Our pre-CS orienting ANOVA found a significant effect of session (*F*_6,120_ = 10.6, *p* < 0.0001) and sex (*F*_1,20_ = 5.71, *p* = 0.027) (figure 2*f*; electronic supplementary material, figure S4*a*–*c*). No effect of condition (*F*_1,20_ = 0.61, *p* = 0.45) and no interactions between condition and sex (*F*_1,20_ = 0.181, *p* = 0.68), sex and session (*F*_6,120_ = 1.4, *p* = 0.22), condition and session (*F*_6,120_ = 1.39, *p* = 0.22), or condition, session and sex (*F*_6,120_ = 0.87, *p* = 0.52) were detected. Our CS1 orienting ANOVA found a significant effect of session (*F*_6,120_ = 6.61, *p* < 0.0001) and significant interactions between sex and session (*F*_6,120_ = 3.39, *p* = 0.004) and sex, condition and session (*F*_6,120_ = 3.34, *p* = 0.004) (figure 2*g*; electronic supplementary material, figure S4*d*–*f*). *Post hoc* testing found a significant difference between males and females (*t* = 3.73, *p* = 0.0003, d = 1.14) and between females with trained and untrained demonstrators (*t* = 2.4, *p* = 0.02, d = 1.33) at session 6 of training only (see electronic supplementary material, figure S4*e*,*f*). No effect of condition (*F*_1,20_ = 0.18, *p* = 0.68), sex (*F*_1,20_ = 1.5, *p* = 0.23), condition and sex interaction (*F*_1,20_ = 1.36, *p* = 0.26), or condition and session interaction (*F*_6,120_ = 0.32, *p* = 0.93) were detected. Finally, our CS2 orienting ANOVA detected a significant effect of session (*F*_6,120_ = 18.502, *p* < 0.0001) and significant interactions between sex and session (*F*_6,120_ = 3.57, *p* = 0.003), condition and session (*F*_6,120_ = 2.37, *p* = 0.034), and condition, sex and session (*F*_6,120_ = 2.29, *p* = 0.04) (figure 2*h*; electronic supplementary material, figure S4*g*–*i*). *Post hoc* testing found a significant effect of condition for males at session 2 (*t*_22_ = 2.068, *p* = 0.045, d = 1.56), session 3 (*t*_22_ = 2.608, *p* = 0.0128, d = 1.43) and session 4 (*t*_22_ = 2.046, *p* = 0.0475, d = 1.38). Significant differences between males and females were also detected at session 6 (*t*_22_ = 2.55, *p* = 0.013, d = 0.828) and a near significant difference at session 7 (Z = 1.88, *p* = 0.06, *r* = 0.383). Testing of the condition by session interaction found a significant effect of condition at session 2 (*t*_22_ = 2.37, *p* = 0.0206, d = 1.26) and a near significant effect at session 4 (*t*_22_ = 1.84, *p* = 0.0696, d = 0.675) for CS2 orienting (see electronic supplementary material, figure S4*g*,*i*). At the CS2 timepoint, no overall effect of sex (*F*_1,20_ = 1.29, *p* = 0.269) or condition (*F*_1,20_ = 0.895, *p* = 0.356) and no interactions between sex and condition (*F*_1,20_ = 1.87, *p* = 0.19) were detected.

#### 3.2.3. Correlational analyses of observer and paired demonstrator behaviour

**Figure 3.**
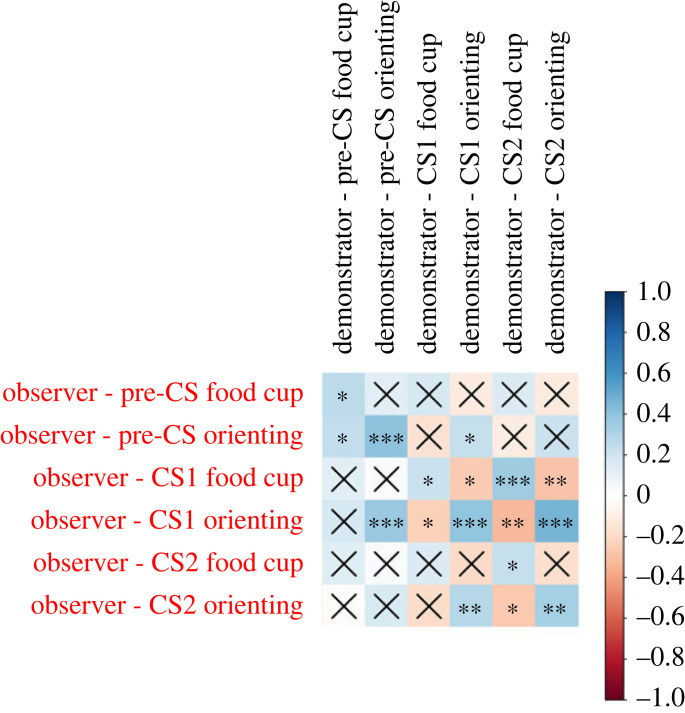
Experiment 2 correlations between demonstrator and observer behaviours. A correlation heat map of observer behaviour across training and the behaviour of their paired demonstrator during the observation phase. Correlations were calculated using averaged demonstrator behaviour across observation sessions and observer behaviour at each observer training session. *p < 0.05, ** p < 0.01, *** p < 0.001*.*

Exploratory analyses of the relationship between observer behaviour over the course of training and the behaviour of their demonstrator were conducted using the demonstrator's orienting and food cup contact scores at each timepoint averaged across all seven observation sessions. As observer behaviour evolved across the course of training, observer behavioural scores were not averaged and, instead, individual session scores were all paired with the averaged behavioural scores obtained from their demonstrator. When interpreting these results, it important to keep in mind that food cup contact and orienting were scored as mutually exclusive behaviours. As such, they were naturally negatively correlated at each timepoint (for observers, across all training sessions: pre-CS: *t*_166_ = −2.74, *p* = 0.0067, *r* = −0.21; CS1: *t*_166_ = −4.9, *p* < 0.0001, *r* = −0.355; CS2: *t*_166_ = −8.79, *p* < 0.0001, *r* = −0.56). Notably, for all analyses here the observer and demonstrator behaviours at CS1 and CS2 were not adjusted for pre-CS responding to avoid obscuring what would otherwise be valid correlations in the case that pre-CS responding was also correlated with a given demonstrator behaviour. To gauge which observer and demonstrator behaviours might be correlated overall, the correlation coefficient and *p*-value for every combination of observer and demonstrator behaviours were calculated and visualized in a correlation heat map (figure 3; specific values can be found in electronic supplementary material, table S1).

**Figure 4.**
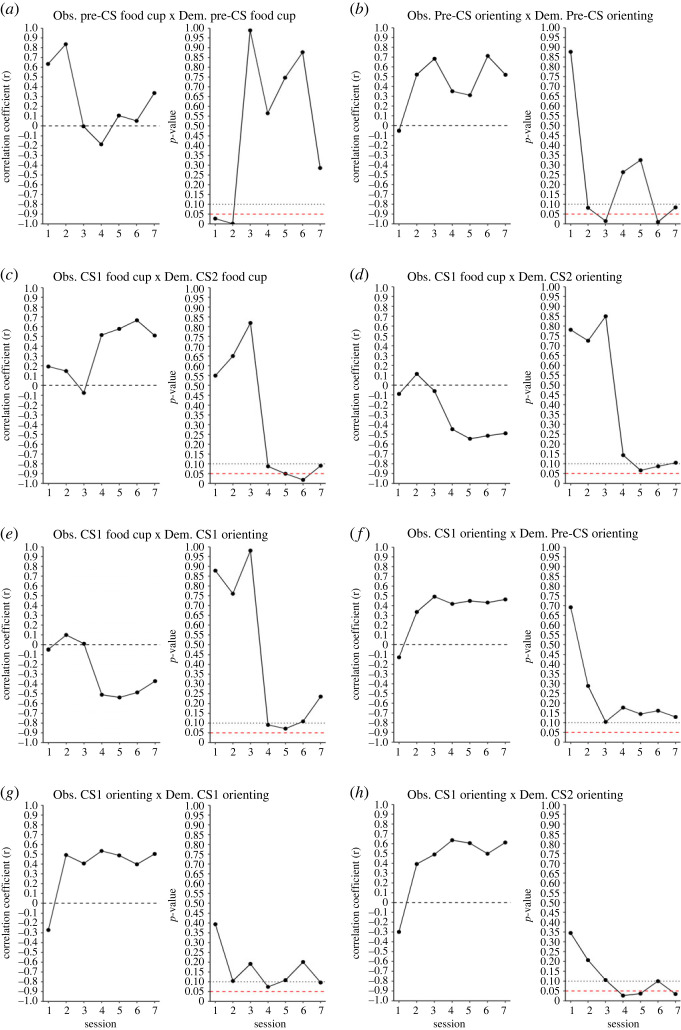
Demonstrator/observer behaviour correlations across sessions. The above figures display the correlation coefficients (left) and associated *p*-values (right) for each session and for each combination of observer/demonstrator behaviours that displayed a trend across sessions. Behaviour combinations are indicated by the title above each correlation/*p*-value plot combination. Dashed black lines (left) indicate a *r* = 0, while the dotted black line (right) and the dashed red line indicate a *p*-value point of 0.1 and 0.05, respectively.

Significant correlations were detected between a number of observer and demonstrator behaviours using this method. However, given the large number of comparisons (36 total), the inflation of sample size by the repetition of demonstrator score at each session and the potential confound of repeated measures in the observer data, further analysis was necessary to determine which (if any) of the observer behaviours were related to their demonstrator's behaviour. In order to do this, the correlations between observer and demonstrator behaviours were calculated for each session and correlation coefficient and *p*-values were plotted by session. Data were then visually assessed by experimenters to identify trends in responding. Paired behaviours determined to probably be related based on correlation patterns across sessions are visualized in figure 4. To provide a rough gauge of how likely each distribution of comparisons was to have occurred by chance, demonstrator scores for the selected behaviour combinations (those shown in figure 4) were scrambled for each behaviour and assigned to a random observer. The correlations between observers and demonstrator behaviours were then recalculated using the scrambled demonstrator data for each session and the sum of the probabilities for each session was taken. This was repeated over 10 000 iterations to create a probability distribution of *p*-values for each of the selected demonstrator/observer behavioural combinations. The probability of obtaining a summed *p*-value less than or equal to the observed value was then calculated. Of the selected behaviour combinations, the only combinations to return a *p* < 0.1 were the observer and demonstrator CS1 orienting combination (*p* = 0.0644) and the observer CS1 orienting and demonstrator CS2 orienting combinations (*p* = 0.0255). Notably, this method is much more likely to support the significance of results in cases which the observer behaviour began correlating with demonstrator behaviour in an early and persistent fashion. As such, these results should not necessarily be taken to mean the correlations between other behavioural combinations are not valid, just that they should be assessed with more caution.

## 4. Discussion

Our investigation found that the experience of having observed multiple sessions of a trained demonstrator responding to the cue was sufficient to alter the behaviour of observers during the individual acquisition process; however, observers were unable to acquire an appetitive association to a neutral light cue through observation of a trained conspecific alone. This was true regardless of whether or not the cue-associated reward was clearly visible to the observer. Interestingly, the behavioural effects of observation largely emerged after the first session of training. This was particularly true when examining behaviours that occurred in the period during which the light CS was illuminated. As such, while some of our results might be explained by mimicry of the demonstrator (e.g. pre-CS food cup responding), not all of the behavioural differences seen between observers with trained demonstrators and observers with no demonstrator/an untrained demonstrator can necessarily be explained by simple mimicry or stimulus enhancement.

### 4.1. Behavioural differences in observers with a trained demonstrator

Significant differences in observer behaviour over the course of training were observed at a few behaviour/timepoint combinations. In experiment 1, greater faceplate contact during the CS1 period was observed in T-Obs rats. This difference did not present itself until session 3 of individual learning and largely persisted through to the end of training. No such separation in performance between rats in the two conditions was observed during the CS2 period when the sucrose solution was actually available for consumption. During a subset of later sessions, we did see some significant differences between conditions in the number of trials in which no sucrose solution was consumed. This was only true when analysing males and females separately, however. In all significant cases, T-Obs rats had fewer missed trials than UT-Obs rats. This would suggest that rats which had a trained demonstrator may have had slightly better task performance. In experiment 2, an overall decrease in pre-CS food cup contact was found in D-Obs rats over ND-Obs rats. This finding provides further evidence that the behaviour of the rats in both these experiments is unlikely to be due to direct mimicry of their demonstrator. Further in support of this idea, D-Obs rats displayed higher overall orienting to the light CS in experiment 2, a difference that only reached significance past session 1.

### 4.2. Sex differences

It is notable that the trend of longer faceplate contact during the CS1 period in experiment 1 seemed to be largely driven by our female subjects (see electronic supplementary material, figure S1*d*–*f* for reference), though this trend was not strong enough to generate a sex by condition interaction in our model. If a legitimate trend, this might be explained by the higher taste sensitivity that female rats display for sucrose [41–43] and the associated enhancement in the acquisition of sucrose-motivated reward learning [44]. Indeed, our own analysis of demonstrator rats during training suggests that females were able to drink significantly more of the sucrose solution than males across training regardless of session number, possibly indicating faster acquisition of the cue–reward association. Our female observers may also have perceived the sucrose solution as a more valuable reward than our males, resulting in increased attention to the goal site in anticipation of the sucrose sipper. It is interesting that this sex difference in consumption was not seen in our observer animals, though whether this lack of an effect was due to observers having less experience with the sucrose solution (i.e. because observers were not allowed 1 h free access to the sucrose solution before training) or due to the observation sessions somehow equalizing the responses of male and female observers across training is unclear. It is also notable that the addition of a conspecific to the conditioning chamber was sufficient to equalize sucrose intake across sexes in demonstrator rats. In experiment 2, some sex differences in orienting behaviours were also observed, with female rats displaying increased orienting to the cue during the pre-CS period in later sessions, while male D-Obs rats displayed increased CS2 period orienting over their ND-Obs counterparts for a longer period of time than did their female D-Obs counterparts.

### 4.3. Relationship between demonstrator behaviour at observation and observer behaviour during training

It is important to note that given the design of our study, statistical analysis of the relationship between observer and demonstrator behaviour without inflating sample size or the probability of Type II errors by repeated testing was difficult. The possibility also remains that those observer rats displaying the highest orienting (and, by extension, generally lower levels of food cup contact) were paired with demonstrators that also happened to display the highest level of orienting by chance. This is a particular threat given our small sample size for correlation analyses (*n* = 12). As such, the results of these analyses should be interpreted with some caution.

Our correlational analysis of demonstrator behaviour during the observation period and the behaviour of their paired observer during training indicated a likely influence of demonstrator responding on observers. Virtually all of the correlations detected in our analysis trended in a direction that suggested that observers were influenced to engage in orienting or food cup contact more if their demonstrator had also engaged more in that behaviour. Notably, food cup behaviour at the CS2 timepoint seemed generally resistant to demonstrator influence. This is probably because food cup contact during this time period was driven primarily by the depositing of food pellets in the cup during the bulk of that period (the last 3 s out of 5 s). When pellets were dispensed into the food cup, sound was made both by the pellet dispenser and by the pellet falling into the jar. These sounds probably drove observer interaction with the food cup or the area around the food cup even in the absence of a learned association and was probably sufficient to obscure any influence demonstrator behaviour might have had on observer responding at the timepoint. In line with this interpretation, both food cup and orienting responding at the CS1 timepoint were correlated with the largest number of demonstrator behaviours and tended to have the higher magnitude correlation coefficients. Future research might attempt to intentionally pair demonstrators that display high orienting with observers and compare later observer behaviour with observers paired with low-orienting demonstrators. If the results of our experiment persist, this model has the potential to serve as a tool for examining social learning of orienting and sign-tracking behaviour.

### 4.4. Reward visibility

Our results indicated that increasing reward visibility was not sufficient to induce observational learning of a cue–reward association. If anything, our results from experiment 1, which used a hidden reward (the sucrose solution) were more suggestive of an influence of demonstrator observation on acquisition of the CS–US association. This is somewhat surprising given the results of Laland & Plotkin [19], who found that the ability of observer rats to learn to dig for carrot pieces through observation of a trained conspecific was contingent on carrot pieces being available to their demonstrator (i.e. on the observer's ability to see the demonstrator rewarded for their digging behaviour). There are a number of possible explanations for why the sucrose solution might have been more effective than the pellets, which were visible. First, as mentioned in the discussion of sex differences, the increase in CS1 faceplate responding seen in T-Obs rats was primarily driven by our female rats for whom the sucrose solution was probably a more motivating reward. Though our rats in experiment 2 were food restricted, this restriction was fairly mild, and the pellets received as reward were quite small and not calorie dense. It is possible that had a more substantial reward been provided observational learning might have occurred. Another possibility is that the sucrose solution was, despite being hidden, a more salient stimulus than the food pellets. While the food pellets were made visible, rat eyesight is poor, and it is unlikely that the grain pellets carried a strong scent. By contrast, it quite is possible that the scent of the sucrose solution was detectable and attractive to the observer. Finally, as the house light remained on for the duration of experiment 1 but not experiment 2, it is possible that the sudden illumination of the chamber by the light CS in experiment 2 impaired observational learning.

### 4.5. Future directions

In future research, we will attempt to expand our analysis through the use of larger sample sizes or a longer observation period. Additionally, we plan to assess whether a higher-value and/or more salient reward (e.g. a sweetened yogurt pellet with a strong scent), observer pre-exposure to the reward to assure that they are aware it is palatable and available from the reward site, or a longer/more intensive period of food restriction might be sufficient to promote social learning. Allowing observer rats a session of individual training or even just individual exposure to the rewarding stimuli in the training context might also be effective in facilitating social learning.

## 5. Conclusion

While our results indicate that rats' behaviour and learning can be influenced by observation of a trained demonstrator, our findings do not suggest that they were able to acquire a cue–reward association via observation of a trained demonstrator alone. This was true both when the reward was clearly visible and when the reward was hidden. Our results do suggest that observation of a conspecific engaging in a given behaviour increases expression of the behaviour later during individual learning. This effect was most apparent when examining orienting. Interestingly, the relationship between observer and demonstrator behaviours was present only past the first session of training. These results seem to indicate that, rather than directly mimicking the behaviour of demonstrators, observers may model their post-learning behaviour on the behaviour of their demonstrator.

## Data Availability

Raw data files are available in The Monfils Lab repository, housed in the Texas Data Repository in Dataverse (https://dataverse.tdl.org/dataverse/MonfilsFearMemoryLab). All other materials are available by request to the authors. Supplementary material is available online [45].
